# Supplementary material for: A Likelihood Approach for Real-Time Calibration of Stochastic Compartmental Epidemic Models
Source: PLoS Comput Biol. 2017 Jan 17;13(1):e1005257. doi: 10.1371/journal.pcbi.1005257 (PMC5240920; doi:10.1371/journal.pcbi.1005257)
Supplement: S1 File — (TAR.GZ) [file pcbi.1005257.s014.tar.gz › HSPH_Online-SI-Revision/PF08/n1-mild/n1-mild_PF2_table-4.pdf]

1	newly	3 weeks	spec	24.	46.31	{16., 88.}	109.125	0.308161	newly	3 weeks	cumul	78.	143.5	{50., 273.}	99.1026	0.266796	newly	5 weeks	spec	32.	39.49	{10., 71.}	59.6563	0.275661	newly	5 weeks	cumul	149.	225.56	{76., 421.}	74.1208	0.238243	newly	59 weeks	cumul	331.	459.6	{190., 735.}	59.4743	0.229993
2	newly	3 weeks	spec	34.	15.11	{0., 38.}	64.5	0.757908	newly	3 weeks	cumul	90.	54.64	{5., 106.}	57.4889	0.527449	newly	5 weeks	spec	17.	12.15	{0., 28.}	63.4706	0.633415	newly	5 weeks	cumul	145.	80.08	{7., 159.}	58.7862	0.57417	newly	55 weeks	cumul	202.	157.45	{8., 386.}	65.6089	0.574489
3	newly	3 weeks	spec	20.	49.27	{2., 104.}	184.45	0.572232	newly	3 weeks	cumul	57.	143.33	{13., 285.}	186.333	0.529005	newly	5 weeks	spec	25.	41.41	{2., 88.}	112.6	0.51378	newly	5 weeks	cumul	111.	229.94	{21., 453.}	146.847	0.503053	newly	64 weeks	cumul	238.	465.64	{55., 781.}	123.941	0.472274
4	newly	3 weeks	spec	15.	40.02	{14., 67.}	175.733	0.419058	newly	3 weeks	cumul	42.	124.55	{50., 211.}	201.167	0.426553	newly	5 weeks	spec	9.	33.44	{12., 67.}	279.778	0.545491	newly	5 weeks	cumul	71.	195.59	{74., 323.}	181.225	0.406902	newly	58 weeks	cumul	398.	422.37	{188., 738.}	41.5854	0.203182
5	newly	3 weeks	spec	50.	29.41	{1., 62.}	52.78	0.560707	newly	3 weeks	cumul	122.	96.77	{5., 182.}	51.2869	0.413763	newly	5 weeks	spec	37.	24.56	{1., 53.}	56.8108	0.549993	newly	5 weeks	cumul	231.	148.69	{10., 283.}	49.2338	0.447225	newly	60 weeks	cumul	369.	302.11	{38., 567.}	47.8076	0.38385
6	newly	3 weeks	spec	26.	47.63	{3., 99.}	126.269	0.507195	newly	3 weeks	cumul	47.	139.27	{14., 277.}	222.447	0.511272	newly	5 weeks	spec	37.	40.14	{3., 85.}	68.2162	0.458003	newly	5 weeks	cumul	121.	223.6	{28., 433.}	121.868	0.424349	newly	64 weeks	cumul	400.	460.95	{61., 766.}	54.3975	0.319744
7	newly	3 weeks	spec	25.	42.41	{4., 88.}	115.32	0.476252	newly	3 weeks	cumul	44.	132.85	{16., 263.}	229.068	0.522661	newly	5 weeks	spec	48.	37.28	{2., 77.}	55.8333	0.522269	newly	5 weeks	cumul	142.	210.59	{32., 407.}	94.1761	0.413751	newly	63 weeks	cumul	381.	433.35	{74., 752.}	56.2546	0.332415
8	newly	3 weeks	spec	13.	24.5	{5., 47.}	113.231	0.375221	newly	3 weeks	cumul	44.	75.1	{28., 128.}	90.1818	0.286233	newly	5 weeks	spec	14.	20.08	{1., 41.}	91.8571	0.465374	newly	5 weeks	cumul	78.	116.91	{32., 199.}	77.7308	0.283285	newly	55 weeks	cumul	449.	267.36	{39., 519.}	48.4365	0.414684
9	newly	3 weeks	spec	32.	22.31	{4., 41.}	50.9063	0.403093	newly	3 weeks	cumul	72.	74.4	{24., 143.}	48.6111	0.245023	newly	5 weeks	spec	42.	17.7	{2., 33.}	62.4286	0.623304	newly	5 weeks	cumul	158.	112.01	{32., 218.}	47.6646	0.325466	newly	54 weeks	cumul	322.	228.97	{38., 414.}	49.7795	0.378163
10	newly	3 weeks	spec	37.	31.06	{12., 53.}	42.0541	0.247071	newly	3 weeks	cumul	113.	100.67	{42., 183.}	40.1858	0.210179	newly	5 weeks	spec	27.	24.18	{7., 43.}	46.8889	0.273817	newly	5 weeks	cumul	184.	153.35	{59., 270.}	40.5054	0.225299	newly	55 weeks	cumul	266.	296.91	{87., 540.}	50.2218	0.235227
11	newly	3 weeks	spec	20.	21.65	{2., 45.}	66.15	0.434034	newly	3 weeks	cumul	44.	70.95	{15., 142.}	91.7045	0.337229	newly	5 weeks	spec	18.	16.63	{1., 33.}	56.3889	0.458968	newly	5 weeks	cumul	86.	107.26	{20., 209.}	67.4186	0.334994	newly	54 weeks	cumul	455.	211.25	{27., 410.}	55.7824	0.554462
12	newly	3 weeks	spec	14.	22.53	{6., 39.}	87.6429	0.332555	newly	3 weeks	cumul	27.	75.28	{26., 135.}	190.	0.438332	newly	5 weeks	spec	18.	19.32	{1., 38.}	57.6667	0.412054	newly	5 weeks	cumul	68.	116.45	{37., 208.}	90.8971	0.30307	newly	55 weeks	cumul	321.	252.34	{41., 473.}	46.5732	0.353185
13	newly	3 weeks	spec	16.	17.61	{1., 39.}	72.5625	0.476036	newly	3 weeks	cumul	60.	59.98	{8., 123.}	59.8	0.377011	newly	5 weeks	spec	15.	13.52	{0., 35.}	75.4667	0.592476	newly	5 weeks	cumul	111.	88.54	{10., 192.}	55.7838	0.430228	newly	56 weeks	cumul	193.	173.41	{12., 364.}	68.6891	0.49743
14	newly	3 weeks	spec	29.	30.91	{0., 66.}	71.8966	0.53877	newly	3 weeks	cumul	93.	104.16	{5., 194.}	72.2151	0.469641	newly	5 weeks	spec	30.	25.11	{0., 56.}	60.1667	0.544018	newly	5 weeks	cumul	159.	158.1	{6., 292.}	63.2704	0.463654	newly	60 weeks	cumul	260.	316.97	{38., 586.}	67.2654	0.417265
15	newly	3 weeks	spec	25.	29.8	{3., 66.}	76.96	0.459662	newly	3 weeks	cumul	71.	95.11	{9., 182.}	84.0704	0.403338	newly	5 weeks	spec	20.	25.17	{1., 56.}	82.55	0.479015	newly	5 weeks	cumul	129.	147.48	{15., 292.}	70.5581	0.386415	newly	60 weeks	cumul	389.	315.25	{38., 606.}	49.5964	0.377566
16	newly	3 weeks	spec	23.	42.48	{17., 76.}	97.3043	0.265626	newly	3 weeks	cumul	52.	132.83	{47., 253.}	159.442	0.356272	newly	5 weeks	spec	7.	34.37	{14., 63.}	393.857	0.635767	newly	5 weeks	cumul	83.	205.75	{88., 364.}	151.289	0.348502	newly	58 weeks	cumul	349.	413.34	{194., 661.}	39.0029	0.159469
17	newly	3 weeks	spec	17.	31.83	{1., 67.}	133.471	0.55519	newly	3 weeks	cumul	43.	99.	{5., 201.}	169.302	0.540948	newly	5 weeks	spec	15.	26.44	{2., 57.}	121.2	0.511985	newly	5 weeks	cumul	78.	154.41	{16., 297.}	138.731	0.496086	newly	61 weeks	cumul	332.	357.79	{45., 661.}	54.8524	0.371219
18	newly	3 weeks	spec	18.	22.68	{6., 40.}	63.4444	0.284254	newly	3 weeks	cumul	52.	76.86	{22., 136.}	73.8077	0.26112	newly	5 weeks	spec	18.	17.73	{4., 34.}	52.2778	0.353466	newly	5 weeks	cumul	99.	113.97	{32., 203.}	53.9899	0.252946	newly	54 weeks	cumul	320.	229.44	{48., 406.}	42.9813	0.32993
19	newly	3 weeks	spec	40.	37.24	{3., 69.}	52.5	0.415405	newly	3 weeks	cumul	125.	116.94	{17., 221.}	52.112	0.368127	newly	5 weeks	spec	35.	33.92	{1., 70.}	64.8	0.519309	newly	5 weeks	cumul	206.	187.63	{22., 332.}	50.1214	0.371149	newly	61 weeks	cumul	389.	392.89	{28., 741.}	54.2699	0.386688
20	newly	3 weeks	spec	37.	40.97	{17., 74.}	49.1622	0.202587	newly	3 weeks	cumul	71.	130.99	{57., 243.}	93.6197	0.24893	newly	5 weeks	spec	33.	33.58	{12., 69.}	52.1212	0.248258	newly	5 weeks	cumul	150.	202.66	{89., 352.}	55.9333	0.186495	newly	58 weeks	cumul	270.	410.42	{209., 733.}	65.1778	0.20656
21	newly	3 weeks	spec	20.	21.91	{0., 48.}	71.35	0.512845	newly	3 weeks	cumul	49.	75.67	{6., 152.}	100.388	0.422986	newly	5 weeks	spec	20.	17.33	{0., 38.}	63.15	0.583087	newly	5 weeks	cumul	108.	112.5	{9., 239.}	64.2222	0.411918	newly	58 weeks	cumul	233.	216.73	{12., 439.}	59.2489	0.452663
22	newly	3 weeks	spec	7.	44.37	{14., 89.}	535.286	0.71222	newly	3 weeks	cumul	28.	134.77	{47., 256.}	381.321	0.59724	newly	5 weeks	spec	13.	38.43	{14., 68.}	201.154	0.439857	newly	5 weeks	cumul	64.	215.3	{78., 399.}	237.969	0.460039	newly	60 weeks	cumul	439.	474.97	{253., 760.}	35.2688	0.159778
23	newly	3 weeks	spec	19.	26.14	{7., 48.}	68.9474	0.283617	newly	3 weeks	cumul	44.	85.74	{38., 138.}	106.5	0.297354	newly	5 weeks	spec	23.	20.52	{3., 35.}	49.3043	0.343791	newly	5 weeks	cumul	99.	130.58	{55., 217.}	59.3131	0.231055	newly	55 weeks	cumul	317.	267.05	{80., 462.}	43.7508	0.293378
24	newly	3 weeks	spec	25.	34.41	{12., 63.}	66.36	0.270679	newly	3 weeks	cumul	65.	106.82	{43., 182.}	80.6154	0.249937	newly	5 weeks	spec	28.	27.92	{7., 52.}	52.	0.326388	newly	5 weeks	cumul	129.	165.44	{65., 297.}	58.0775	0.229055	newly	57 weeks	cumul	399.	347.35	{88., 569.}	39.0501	0.254592
25	newly	3 weeks	spec	15.	23.64	{2., 48.}	95.4667	0.397333	newly	3 weeks	cumul	35.	76.31	{17., 154.}	140.2	0.376113	newly	5 weeks	spec	9.	18.79	{0., 41.}	153.	0.581804	newly	5 weeks	cumul	63.	115.92	{19., 241.}	113.778	0.360733	newly	54 weeks	cumul	293.	229.89	{22., 487.}	56.3652	0.443368
26	newly	3 weeks	spec	28.	35.89	{11., 64.}	62.75	0.255121	newly	3 weeks	cumul	64.	110.83	{42., 201.}	87.8594	0.255099	newly	5 weeks	spec	24.	31.37	{7., 55.}	64.2083	0.285822	newly	5 weeks	cumul	119.	177.38	{62., 297.}	70.3193	0.234495	newly	57 weeks	cumul	217.	385.15	{99., 618.}	95.3779	0.299849
27	newly	3 weeks	spec	24.	24.31	{3., 46.}	56.875	0.407976	newly	3 weeks	cumul	69.	76.12	{15., 142.}	55.6232	0.300814	newly	5 weeks	spec	20.	19.16	{1., 42.}	55.7	0.412538	newly	5 weeks	cumul	118.	117.03	{22., 228.}	51.2797	0.319206	newly	54 weeks	cumul	267.	247.78	{29., 498.}	51.9176	0.378969
28	newly	3 weeks	spec	11.	42.41	{13., 84.}	289.545	0.510277	newly	3 weeks	cumul	24.	125.51	{40., 244.}	422.958	0.629918	newly	5 weeks	spec	16.	37.32	{9., 72.}	149.625	0.381885	newly	5 weeks	cumul	66.	202.11	{67., 385.}	209.803	0.420196	newly	60 weeks	cumul	343.	455.75	{181., 759.}	53.4781	0.20696
29	newly	3 weeks	spec	36.	36.9	{14., 64.}	45.1111	0.225502	newly	3 weeks	cumul	105.	119.32	{42., 208.}	46.5524	0.195163	newly	5 weeks	spec	22.	29.93	{11., 57.}	65.0455	0.247884	newly	5 weeks	cumul	167.	181.33	{73., 335.}	44.497	0.194301	newly	57 weeks	cumul	288.	366.21	{156., 608.}	52.6354	0.206119
30	newly	3 weeks	spec	28.	37.04	{2., 79.}	84.9286	0.498867	newly	3 weeks	cumul	83.	114.32	{5., 234.}	87.0361	0.429028	newly	5 weeks	spec	27.	31.27	{0., 67.}	73.1481	0.531433	newly	5 weeks	cumul	148.	180.68	{12., 353.}	75.027	0.427992	newly	61 weeks	cumul	335.	380.74	{24., 711.}	59.3731	0.392027
31	newly	3 weeks	spec	56.	27.57	{2., 62.}	58.7679	0.621073	newly	3 weeks	cumul	151.	89.19	{8., 177.}	52.3377	0.495835	newly	5 weeks	spec	34.	21.94	{1., 50.}	58.5882	0.60687	newly	5 weeks	cumul	244.	135.65	{12., 301.}	53.9139	0.504856	newly	59 weeks	cumul	353.	302.18	{33., 584.}	52.5439	0.399786
32	newly	3 weeks	spec	11.	33.25	{5., 69.}	221.	0.515427	newly	3 weeks	cumul	27.	107.31	{32., 198.}	304.852	0.541678	newly	5 weeks	spec	24.	26.66	{2., 53.}	61.6667	0.41288	newly	5 weeks	cumul	76.	163.94	{33., 318.}	133.895	0.361309	newly	57 weeks	cumul	391.	329.87	{33., 567.}	40.9437	0.301832
33	newly	3 weeks	spec	37.	38.93	{14., 69.}	51.4865	0.237242	newly	3 weeks	cumul	84.	124.31	{51., 211.}	67.369	0.211263	newly	5 weeks	spec	32.	31.64	{11., 57.}	50.125	0.255872	newly	5 weeks	cumul	159.	192.04	{80., 320.}	53.4717	0.204999	newly	58 weeks	cumul	332.	411.23	{146., 692.}	50.0753	0.202786
34	newly	3 weeks	spec	13.	35.21	{14., 70.}	177.154	0.388797	newly	3 weeks	cumul	35.	111.61	{51., 195.}	219.343	0.444932	newly	5 weeks	spec	9.	30.48	{8., 58.}	248.889	0.50596	newly	5 weeks	cumul	63.	174.59	{74., 310.}	179.571	0.389504	newly	57 weeks	cumul	230.	370.95	{124., 654.}	83.4217	0.269351
35	newly	3 weeks	spec	24.	45.26	{13., 92.}	106.833	0.291397	newly	3 weeks	cumul	77.	136.44	{48., 262.}	93.2987	0.26571	newly	5 weeks	spec	30.	36.07	{12., 70.}	57.1667	0.23007	newly	5 weeks	cumul	154.	212.8	{80., 425.}	64.3377	0.223026	newly	59 weeks	cumul	405.	442.2	{204., 717.}	37.4173	0.168244
36	newly	3 weeks	spec	51.	36.32	{3., 70.}	51.1765	0.491229	newly	3 weeks	cumul	141.	113.3	{9., 233.}	51.2908	0.411647	newly	5 weeks	spec	46.	31.15	{3., 62.}	53.1957	0.528262	newly	5 weeks	cumul	242.	179.53	{17., 347.}	48.5661	0.419045	newly	61 weeks	cumul	339.	382.75	{70., 679.}	54.4454	0.339515
37	newly	3 weeks	spec	18.	35.1	{14., 60.}	106.778	0.311487	newly	3 weeks	cumul	52.	107.72	{46., 174.}	113.115	0.294712	newly	5 weeks	spec	33.	30.38	{10., 56.}	45.3333	0.289337	newly	5 weeks	cumul	122.	170.05	{73., 294.}	56.9754	0.196356	newly	57 weeks	cumul	268.	374.52	{152., 609.}	61.4104	0.225595
38	newly	3 weeks	spec	43.	37.29	{3., 69.}	51.2326	0.443295	newly	3 weeks	cumul	95.	115.07	{6., 219.}	68.2842	0.38875	newly	5 weeks	spec	60.	33.12	{0., 70.}	56.2667	0.653243	newly	5 weeks	cumul	220.	184.79	{16., 332.}	48.5409	0.401966	newly	61 weeks	cumul	371.	390.83	{22., 741.}	56.9569	0.403546
39	newly	3 weeks	spec	35.	26.38	{3., 59.}	54.8571	0.457582	newly	3 weeks	cumul	95.	86.16	{10., 167.}	55.9789	0.374942	newly	5 weeks	spec	16.	20.3	{0., 44.}	85.75	0.515183	newly	5 weeks	cumul	151.	129.96	{12., 256.}	54.5828	0.377342	newly	59 weeks	cumul	278.	270.52	{33., 543.}	56.5971	0.366295
40	newly	3 weeks	spec	40.	37.9	{3., 78.}	56.45	0.443442	newly	3 weeks	cumul	91.	112.46	{16., 241.}	72.7692	0.414372	newly	5 weeks	spec	51.	34.35	{2., 69.}	54.2157	0.571046	newly	5 weeks	cumul	193.	183.79	{25., 367.}	55.1036	0.428823	newly	62 weeks	cumul	329.	395.98	{44., 736.}	67.5684	0.430861
41	newly	3 weeks	spec	26.	38.75	{3., 86.}	99.2692	0.471318	newly	3 weeks	cumul	100.	126.83	{15., 254.}	81.69	0.417522	newly	5 weeks	spec	38.	34.23	{2., 74.}	62.5526	0.531406	newly	5 weeks	cumul	177.	199.15	{25., 402.}	69.339	0.408697	newly	63 weeks	cumul	284.	421.63	{70., 761.}	85.6232	0.394135
42	newly	3 weeks	spec	58.	40.34	{3., 78.}	48.4138	0.467162	newly	3 weeks	cumul	126.	117.22	{7., 231.}	54.5556	0.423012	newly	5 weeks	spec	44.	38.1	{2., 77.}	57.0455	0.492122	newly	5 weeks	cumul	229.	194.42	{18., 355.}	48.3668	0.427323	newly	62 weeks	cumul	291.	449.78	{58., 795.}	88.1856	0.441235
43	newly	3 weeks	spec	61.	45.81	{3., 99.}	53.459	0.476507	newly	3 weeks	cumul	155.	133.51	{8., 278.}	55.8903	0.449539	newly	5 weeks	spec	49.	39.92	{3., 84.}	53.9184	0.461704	newly	5 weeks	cumul	274.	216.96	{21., 427.}	50.9124	0.440459	newly	64 weeks	cumul	392.	485.38	{68., 783.}	56.8776	0.361927
44	newly	3 weeks	spec	34.	31.64	{8., 55.}	49.7059	0.344598	newly	3 weeks	cumul	64.	102.31	{35., 173.}	81.1094	0.267844	newly	5 weeks	spec	35.	24.66	{3., 49.}	51.5429	0.438181	newly	5 weeks	cumul	143.	154.96	{50., 285.}	51.6364	0.25696	newly	56 weeks	cumul	211.	301.32	{70., 532.}	76.1422	0.318793
45	newly	3 weeks	spec	33.	24.97	{7., 45.}	45.4242	0.295267	newly	3 weeks	cumul	81.	79.96	{32., 143.}	42.7901	0.206631	newly	5 weeks	spec	24.	19.86	{4., 36.}	49.	0.387007	newly	5 weeks	cumul	141.	120.48	{51., 220.}	41.8014	0.236312	newly	54 weeks	cumul	301.	259.1	{62., 496.}	47.5681	0.300098
46	newly	3 weeks	spec	19.	31.35	{1., 63.}	115.105	0.560549	newly	3 weeks	cumul	57.	98.14	{5., 204.}	120.14	0.500198	newly	5 weeks	spec	23.	26.58	{3., 56.}	72.6087	0.4683	newly	5 weeks	cumul	104.	153.9	{10., 297.}	97.7308	0.457795	newly	61 weeks	cumul	451.	365.42	{70., 638.}	40.2439	0.331086
47	newly	3 weeks	spec	5.	36.08	{14., 71.}	624.4	0.798146	newly	3 weeks	cumul	19.	111.49	{45., 204.}	486.789	0.696031	newly	5 weeks	spec	12.	33.91	{14., 62.}	190.75	0.445652	newly	5 weeks	cumul	47.	180.43	{80., 322.}	283.894	0.520294	newly	59 weeks	cumul	335.	428.05	{195., 702.}	48.1104	0.186825
48	newly	3 weeks	spec	15.	23.29	{8., 45.}	79.9333	0.271505	newly	3 weeks	cumul	52.	75.15	{31., 134.}	65.9808	0.229775	newly	5 weeks	spec	38.	18.73	{3., 36.}	55.9737	0.527515	newly	5 weeks	cumul	137.	115.22	{36., 218.}	40.9489	0.244817	newly	54 weeks	cumul	271.	252.87	{57., 467.}	48.9557	0.303257
49	newly	3 weeks	spec	12.	34.21	{11., 72.}	193.083	0.430686	newly	3 weeks	cumul	36.	107.66	{43., 237.}	203.056	0.41689	newly	5 weeks	spec	11.	29.14	{6., 58.}	179.636	0.472889	newly	5 weeks	cumul	67.	167.86	{64., 359.}	157.164	0.363449	newly	58 weeks	cumul	292.	367.65	{133., 639.}	54.387	0.23777
50	newly	3 weeks	spec	24.	25.7	{6., 50.}	54.	0.313289	newly	3 weeks	cumul	70.	86.49	{27., 156.}	58.0714	0.253507	newly	5 weeks	spec	19.	19.41	{2., 35.}	57.6316	0.405271	newly	5 weeks	cumul	120.	128.32	{39., 237.}	49.2667	0.257787	newly	55 weeks	cumul	321.	265.6	{44., 469.}	45.2835	0.333828
